# 

**Published:** 1908-04-15

**Authors:** 


### ANNOUNCEMENTS.

SOUTHERN BRANCH OF THE NATIONAL DENTAL ASSOCIATION.—The desire has been expressed by the members of the Southern Branch of the National Dental Association to have the clinics be the leading feature of the meeting in Birmingham, Ala., May 12th to 15th, 1908.

You are especially invited to give a clinic on any subject that would be of interest to the profession.

Every convenience will be furnished the clinician, such as chairs, tables, linen, gas, electricity, etc., he being expected to furnish his own instruments and material and a written report of his clinic, which will be read and discussed before an open session of the Association following the clinics.

Admission to clinic room will be by card or badge, which will be issued to all ethical practitioners in attendance.

The following sub-division of the clinic committee has been made for the more effectual working of its plans:

Dr. H. Clay Hassell, Tuscaloosa, Ala., Chairman.

Dr. T. P. Hinman, Atlanta, Ga., for District of Columbia, Maryland, Delaware, and all territory not in Southern Branch.

Dr. A. J. Cottrell, Knoxville, Tenn., for Tennessee, South Carolina and Georgia.

Dr. I. B. Howell, Paducah, Ky., for Kentucky, Arkansas, and Mississippi.

Dr. B. C. Fowlkes, Selma, Ala., for Alabama, Texas and Oklahoma.

Dr. W. D. Carmichael, Birmingham, Ala., Local Arrangements.

Please write to the chairman or the committeeman of your division at once, or not later than April 10th, giving subject of your clinic, and other information, so that it may be properly inserted in the program, and space allotted for your chair or table.

It is our purpose to have program published in the journals if replies are prompt.

There is every prospect of a large attendance. Will you help make the clinics notable?

H. Clay Hazzell,
Chairman Clinic Committee.

---

The Dental Manufacturers' Exhibit at Madison Square Garden, New York.—The attendance at the recent Dental Manufacturers' Exhibit at Madison Square Garden, New York, was beyond all precedent and broke all records for attendance at dental exhibits. The exhibits were varied, and the great number of new appliances on view was evidence of the decided advancement made in late years by dental manufacturers. This exhibit, representing the principal manufacturers of the United States, marks an era of progress and perfection, and shows that all the requirements for every branch of dental practice are properly and generously supplied. New mechanical contrivances predominated and some devices showing improvement over old methods were shown.

Many Cast Gold Inlay Devices and accessories were shown, although it remains for the profession to decide on the correct and effective outfit. A new vulcanizer with improved closing device was interesting.

Porcelain Teeth and general dental supplies of all kinds were in profusion, as well as many new and old electrical appliances, furniture, chairs, etc.

It is questionable if this form of dealers exhibit would not be better than the usual exhibits at dental societies.

---

THE KENTUCKY STATE DENTAL ASSOCIATION will hold its business sessions at Louisville, June 2nd and 3rd. The regular program will be co-operative with the Semi-Centennial Jubilee meeting at Indianapolis, June 4, 5, 6, 1908.

W. M. RANDALL, Secretary.

---

THE NORTHERN OHIO DENTAL ASSOCIATION.—The fifty-first annual meeting of the Northern Ohio Dental Association will be held at Canton, Ohio, May 26, 27, 28, 1908.

The session's will be held in the citys auditorium, one of the largest in the Middle West, with headquarters at the Courtland Hotel. There are numerous other hotels in Canton, so there will be accomodations for all. Hotel rates may be had at $1.50 to $5.00 per day, American plan.

Canton is essentially a dental manufacturing town, having three large and busy plants. The exhibits will be first class.

The committees are sparing no time nor expense to make this an especially attractive meeting. The program will be up to the standard of previous years. Men of international reputation have been secured to read papers and give clinics.

Remember the time and place, May 26, 27, 28, 1908, Canton, Ohio.

W. H. Whitslar,
J. H. Wible,
F. M. Castro,
Executive Committee.

---

The Virginia State Dental Association.—The 39th annual session of the Virginia State Dental Association will be held at the Main Hall of the Medical College of Virginia, Richmond, Va., beginning July 14th, 1908.

The intention of the Society is to make this the most successful meeting in the history of the organization,

Clinics will be given and papers read by eminent members of the profession.

All ethical practitioners are cordially invited to attend.

W. H. Pearson, Secretary.

---

Illinois State Dental Society.—The forty-fourth annual meeting of the Illinois State Dental Society will be held at Springfield, May 12th, 13th, 14th, 15th, 1908.

The sessions and clinics will be held in the State Armory Building. Wednesday and Friday mornings will be devoted to clinics, other sessions to papers and discussions.

Arthur D. Black, Secretary.

INDIANA STATE DENTAL ASSOCIATION.—The Semi-Centennial Jubilee Meeting of the Indiana State Dental Association at Indianapolis, June 4, 5, 6, will have for essayists the following well known men:

Dr. Truman W. Brophy, Chicago, Ills., Subject: "The deformity of cleft palate; its influence physically and mentally." Dr. George Zederbaum, Charlotte, Mich., Subject: "The education of a nation. Teeth." Dr. H. H. Holmes, Louisville, Ky., Subject: "The influence and benefits of Associations." Dr. M. H. Fletcher, Cincinnati, O., Subject: "Alveolitis; the disease of which Pyorrhea Alveolaris is one stage." Dr. G. V. Black, Chicago, Ills., Subject: "Supernumerary Teeth."

Respectfully,

D. A. HOUSE.

